# Economic effects of dietary salt reduction policies for cardiovascular disease prevention in Japan: a simulation study of hypothetical scenarios

**DOI:** 10.3389/fnut.2023.1227303

**Published:** 2023-11-09

**Authors:** Nayu Ikeda, Hitomi Yamashita, Jun Hattori, Hiroki Kato, Nobuo Nishi

**Affiliations:** ^1^International Center for Nutrition and Information, National Institute of Health and Nutrition, National Institutes of Biomedical Innovation, Health and Nutrition, Settsu, Osaka, Japan; ^2^Department of Healthcare Information Management, The University of Tokyo Hospital, Bunkyo-ku, Tokyo, Japan; ^3^Graduate School of Public Health, St. Luke’s International University, Chuo-ku, Tokyo, Japan

**Keywords:** salt intake, cardiovascular disease, economic evaluation, simulation, healthcare costs

## Abstract

**Objective:**

Reducing dietary salt intake is an essential population strategy for cardiovascular disease (CVD) prevention, but evidence on healthcare costs and outcomes is limited in Japan. We aimed to conduct a pilot economic evaluation under hypothetical scenarios of applying the salt reduction policies of England to Japan.

**Methods:**

We examined salt reduction policies in England: media health promotion campaigns, front-of-pack labeling, and voluntary and mandatory reformulation with best-case and worst-case policy cost scenarios. We assumed that these policies were conducted in Japan for 10 years from 2019. We used published data on epidemiology and healthcare expenditures in Japan and the costs and effects of salt reduction policies in England, and defined the benefits as a decrease in national medical expenditures on CVD. We developed a Markov cohort simulation model of the Japanese population. To estimate the annual net benefits of each policy over 10 years, we subtracted monitoring and policy costs from the benefits. We adopted a health sector perspective and a 2% discount rate.

**Results:**

The cumulative net benefit over 10 years was largest for mandatory reformulation (best case) at 2,015.1 million USD (with costs of USD 48.3 million and benefits of USD 2063.5 million), followed by voluntary reformulation (net benefit: USD 1,895.1 million, cost: USD 48.1 million, benefit: USD 1,943.2 million), mandatory reformulation (worst case, net benefit: USD 1,447.9 million, cost: USD 1,174.5 million, benefit: USD 2,622.3 million), labeling (net benefit: USD 159.5 million, cost: USD 91.6 million, benefit: USD 251.0 million), and a media campaign (net benefit: USD 140.5 million, cost: USD 110.5 million, benefit: USD 251.0 million). There was no change in the superiority or inferiority of policies when the uncertainty of model parameters was considered.

**Conclusion:**

Mandatory reformulation with the best-case cost scenario might be economically preferable to the other alternatives in Japan. In future research, domestic data on costs and effects of salt reduction policies should be incorporated for model refinement.

## Introduction

Cardiovascular disease (CVD) is the major source of healthcare expenditure worldwide (1–3). Excessive dietary salt intake increases the risk of CVD through elevated blood pressure, and it ranks among the top dietary risk factors for premature deaths and disability from CVD in many countries (4, 5). A reduction in population-wide salt consumption is promoted as one of the most cost-effective interventions for primary prevention of hypertension and CVD (6, 7). Lowering salt intake is expected to save the substantial healthcare costs associated with CVD events.

To assess the costs and effectiveness of dietary salt reduction policies, economic modeling studies have been conducted across or within countries (8–17). For example, in England, average salt intake has decreased considerably, partly owing to national salt reduction policies that have been conducted since the 2000s, including a national media campaign, voluntary and mandatory reformulation of processed foods, and front-of-pack labeling (18). Health economic evaluation studies have shown that these policies gained life-years and saved healthcare costs on CVD (19–21).

In Japan, mean salt intake in adults has decreased over the long term (22), but it remains high compared with other countries (23), at 10 g/day in 2019 (24). Heart disease and stroke accounted for approximately 22% of total deaths in 2021 (25), and CVD was responsible for 19% of national healthcare expenditure in 2020 (26). A few dietary salt reduction goals have been recommended to prevent CVD and attenuate the rise in national healthcare costs (27–29). However, the impact of salt reduction policies in Japan is poorly understood, partly because of the lack of accumulated data on program costs and effects. There has been only one simulation study conducted on future health and economic effects of salt reduction policies in Japan (30).

As part of the development of methods for health economic evaluations of nutrition policies in Japan, we aimed to create a simulation model on the costs and benefits of hypothetical scenarios involving the implementation of the salt reduction policies used in England. To build a Japanese prototype model, we utilized published data on policy costs and effects in England, and salt intake, CVD epidemiology, and healthcare costs in Japan.

## Materials and methods

### Salt reduction policy scenarios

We adopted four salt reduction policy scenarios examined in a previous study in England (20): a national media campaign; front-of-pack labeling of processed foods using a traffic light system; voluntary reformulation of processed foods by the food industry; and mandatory reformulation of processed foods with legislation. Details of these policies have been described elsewhere (18, 20). Briefly, the national media campaign was called Change4Life and utilized various media tools to improve public awareness of physical activity and healthy eating, including reducing consumption of foods high in salt. For front-of-pack labeling, food manufacturers and retailers were required to use a standardized traffic light system on all food packaging to inform consumers of the levels of fats, sugars, and salt in processed foods. In voluntary reformulation, the Food Standards Agency established salt targets for the reformulation of food products to be achieved by the food industry on a voluntary basis. In mandatory reformulation, legislation was imposed on food manufacturers to reduce salt contents in processed foods.

### Modeling framework

We modeled the effects of the four salt reduction policies on the incidence and mortality of CVD and national healthcare expenditures. We defined CVD (I00–I99) by the International Classification of Diseases, 10th Revision codes. We developed a discrete-time Markov cohort macro-simulation model using TreeAge Pro Healthcare 2021 (TreeAge Software, Williamstown, Massachusetts, United States).

We obtained data for the input parameters from published survey reports, publicly available databases, and previous studies (Table 1). We used mean dietary salt intake published from the Japan National Health and Nutrition Survey in 2019 (24). This survey employed a stratified two-stage cluster sample design to ensure a nationally representative sample of the non-institutionalized Japanese population (38). Household representatives reported food intake of household members using a 1-day semi-weighted household dietary record. The Standard Tables of Food Composition in Japan 2015 (39) was applied to calculate salt intake of participants from their food intake records.

**Table 1:** Input parameters and data sources used in the simulation model.

Input parameters	Data sources	Values^b^
Total population, all ages	Population Estimates, 2019 (31)	126,197,000 persons
Total deaths, all ages	Vital Statistics, 2019 (32)	1,381,093 deaths
Mean dietary salt intake, age ≥ 20 years	National Health and Nutrition Survey in Japan, 2019 (24)	10.1 g/day
CVD incidence rate, all ages	Global Burden of Disease Study 2019 (33)	1,203 (1,128–1,283) per 100 K
CVD prevalence rate, all ages	Global Burden of Disease Study 2019 (33)	13,500 (12,956–14,064) per 100 K
CVD mortality rate, all ages	Global Burden of Disease Study 2019 (33)	291 (231–326) per 100 K
Changes associated with increases in daily sodium intake		
CVD incidence	Literature (34)	6 (1–11) per 1 g
CVD mortality	Literature (35)	1 (0.2–1.7) per 10 mmol
National medical expenditure^a^	Estimates of National Medical Care Expenditure, 2019 (36)	
Inpatient care		34,549,123,934 USD
Outpatient care		21,747,546,097 USD
Expected reduction of salt intake over 10 years	Literature (20)	
National media campaign		2% (range, 1–5)
Front-of-pack traffic light labeling		2% (range, 1–5)
Voluntary reformulation		15% (range, 5–20)
Mandatory reformulation		20% (range, 10–32)

a The expenditures were converted from Japanese yen to USD according to the annual average exchange rate in 2019 (109.01 JPY per USD) (37).

b Values in parentheses indicate the lower and upper bounds of 95% confidence intervals unless otherwise noted.

CVD, cardiovascular diseases.

We simulated a closed cohort of the total population of all ages in Japan in 2019 over 10 years. We selected 2019 as the baseline year for the simulation because it was the most recent year for which all required data were available for the input parameters. We limited the time horizon to 10 years to ensure the robustness of the study results, given the potential long-term social changes that might occur. We divided the simulation period into annual cycles and conducted the simulation from the perspective of the health sector.

**Figure 1:**
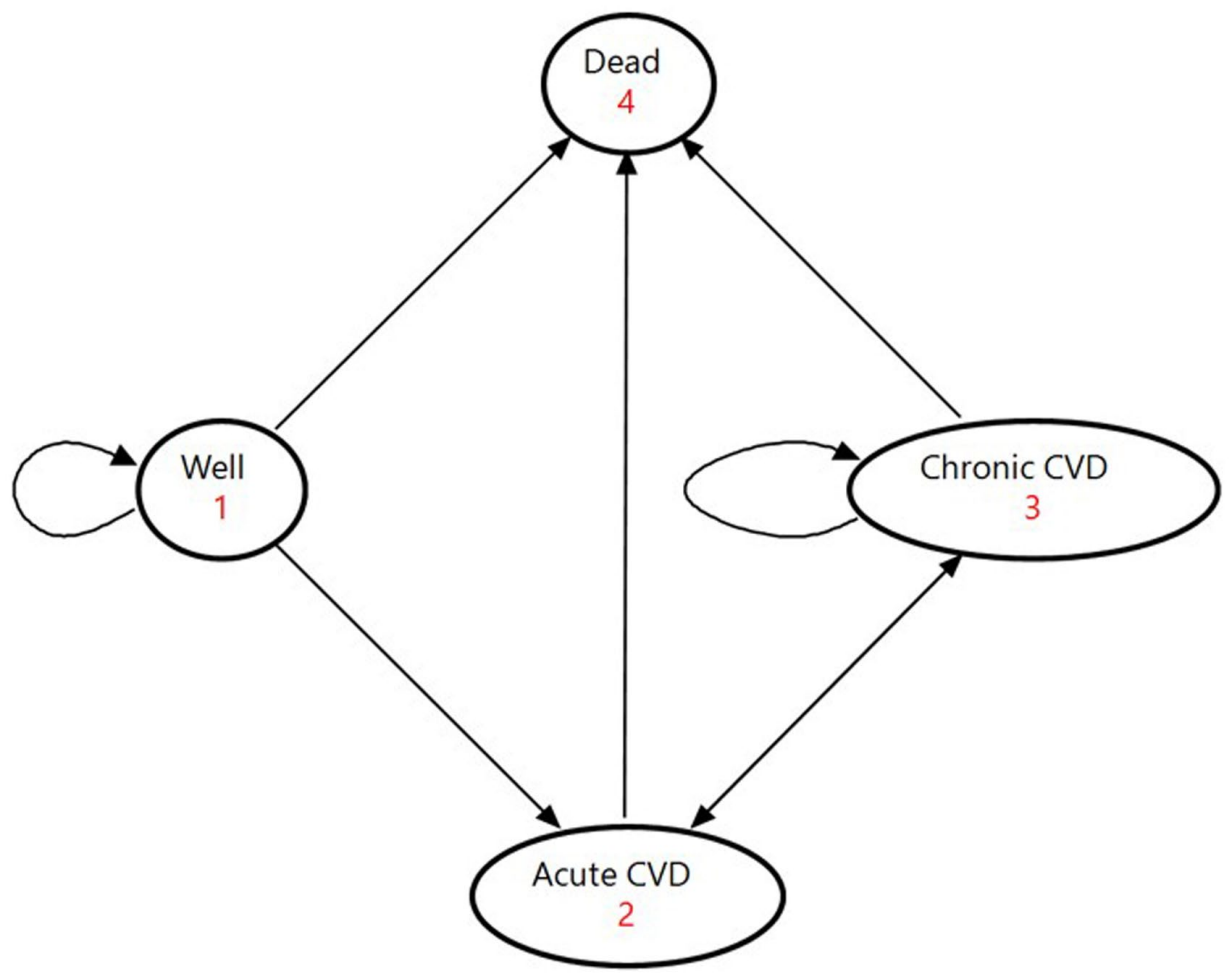
State-transition diagram of the Markov model. The ovals represent the four health states. The direct arrows represent the directions of the transitions of the cohorts between health states. Circular arrows indicate the cohort remaining in each health state. Each arrow has a transition probability. CVD denotes cardiovascular disease.

The model consisted of four mutually exclusive health states: being healthy, acute CVD, chronic CVD, and death (Figure 1). The healthy state represented people who had no history of CVD. The acute state covered people who were admitted to hospital for CVD. The chronic state comprised survivors who continued treatment on an outpatient basis. The death state was an absorbing terminal state. We assigned national healthcare expenditures for inpatient care to the acute state and those for outpatient care to the chronic state.

At the beginning of the Markov process, the cohort members were split among the three states of being healthy, acute CVD, and chronic CVD on the basis of proportions determined by the incidence and prevalence of CVD (Supplementary Table S1). In each successive year, they moved among the four health states according to annual transition probabilities. We calculated the initial values of transition probabilities based on Supplementary Table S2. People in the healthy state either developed a first-ever CVD event, died from causes other than CVD, or maintained good health; they could not return to the healthy state after leaving with an occurrence of CVD. Acute patients moved to the chronic state if they survived to discharge for continuing outpatient care, or they entered the state of death if they died from the disease. Outpatients in the chronic state either experienced a recurrent event, died from other causes, or stayed in the same condition. We obtained transition probabilities by reviewing previous studies for CVD recurrence rates (40–44) and mortality from non-CVD causes among CVD patients (45–47).

The transition probabilities changed over time through the effects of salt reduction policies on the incidence and mortality of CVD. We followed the previous study (20) in setting the magnitude of the effects of policies on mean dietary salt intake over a decade (Table 1). Supposing that mean dietary salt intake decreased annually at a constant rate, we calculated annual reduction rates of mean dietary salt intake at 0.2% for the national media campaign and front-of-pack labeling, 1.5% for voluntary reformulation, and 2.0% for mandatory reformulation. To quantify changes in the incidence and mortality of CVD through decreased dietary salt intake, we applied relative risks estimated by meta-analyses (34, 35) (see Table 1). Mean dietary salt intake and cumulative risk reductions to be achieved over time are provided in Supplementary Table S3.

We made two assumptions to simplify the model according to previous studies. The first assumption was that mortality rates from other causes were equal among the healthy and chronic states (48). The probability of dying from non-CVD events was the same across individuals, regardless of whether or not they had ever had a CVD event. The second assumption is that the full effect of dietary salt reduction on CVD was achieved immediately, without any phase-in period, and last for the rest of the period (9, 10).

### Costs and benefits

We defined the costs of interventions as the sum of policy costs and monitoring costs according to the previous study (20) (Supplementary Table S4). As it is difficult to estimate the exact cost of reformulation outside of natural product cycles for food manufacturers, we considered a best-case and worst-case cost for mandatory reformulation (20). The best-case cost assumes that there is no policy cost incurred to the industry and is equivalent to the cost for voluntary reformulation. The worst-case policy cost for mandatory reformulation was estimated by multiplying the average cost of reducing salt in processed food products (25,000 British pound sterling, GBP) by the target of 20,000 product lines. The policy cost for labeling was estimated as a product of the average cost of a stock control unit (GBP 1,000) and 20,000 product lines needed to change labels to a signaling system. We doubled the sum of the policy and monitoring costs because the total population of Japan (126,167,000 as of October 1, 2019 (31)) was approximately twice that of England and Wales (59,439,840 in mid-2019 (49)). Then, we divided the doubled total costs by 10 to obtain annual total costs, assuming that they were constant during the simulation period.

We defined the benefits of the interventions as a decrease in national healthcare expenditures for CVD through reductions in dietary salt intake. We estimated net benefits by subtracting costs from benefits. We compared the net benefits accumulated over 10 years among the four scenarios to determine a dominant policy. We converted the annual total costs in GBP and national healthcare expenditures in Japanese yen to US dollars (USD) according to the annual average exchange rates in 2019 published by the International Monetary Fund (GBP 0.783 per USD and 109.01 yen per USD) (37). We discounted both costs and benefits at 2% annually, according to the guidelines for economic evaluation of healthcare technologies in Japan (50).

### Sensitivity analysis

We conducted multiple deterministic one-way sensitivity analyses to assess the impact of uncertainty surrounding model input parameters on the net benefits. The parameters examined in the sensitivity analyses were the discount rate (0–4%), the effects of policy interventions, and the incidence, prevalence, mortality, and relative risks of CVD (95% confidence intervals and ranges in Table 1).

## Results

### Costs, benefits, and net benefits of salt reduction policies

**Figure 2:**
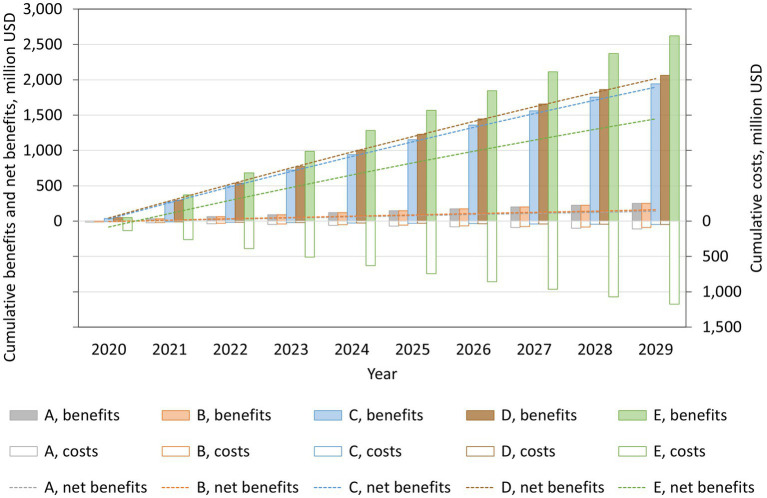
Projected cumulative costs, benefits, and net benefits from reducing mean dietary salt intake, by policy, for the period 2019–2029, in a closed cohort of the population of all ages in 2019. A: national media campaign; B: front-of-pack traffic light labeling; C: voluntary reformulation; D: mandatory reformulation with the best-case policy cost; E: mandatory reformulation with the worst-case policy cost.

The projected costs, benefits, and net benefits of each salt reduction policy are shown in Figure 2. The cumulative 10-year cost was lowest for voluntary reformulation (USD 48.1 million), followed by mandatory reformulation with the best-case cost (USD 48.3 million), labeling (USD 91.6 million), a national media campaign (USD 110.5 million), and mandatory reformulation with the worst-case cost (USD 1,174.5 million). The cumulative 10-year benefit was largest for mandatory reformulation with the worst-case cost (USD 2,622.3 million), followed by mandatory reformulation with the best-case cost (USD 2,063.5 million), voluntary reformulation (USD 1,943.2 million), the national media campaign, and labeling (USD 251.0 million each). The cumulative net benefit was positive for all policies by the end of the simulation period. It was largest for mandatory reformulation with the best-case cost (USD 2,015.1 million), followed by voluntary reformulation (USD 1,895.1 million), mandatory reformulation with the worst-case cost (USD 1,447.9 million), labeling (USD 159.5 million), and the national media campaign (USD 140.5 million).

**Figure 3:**
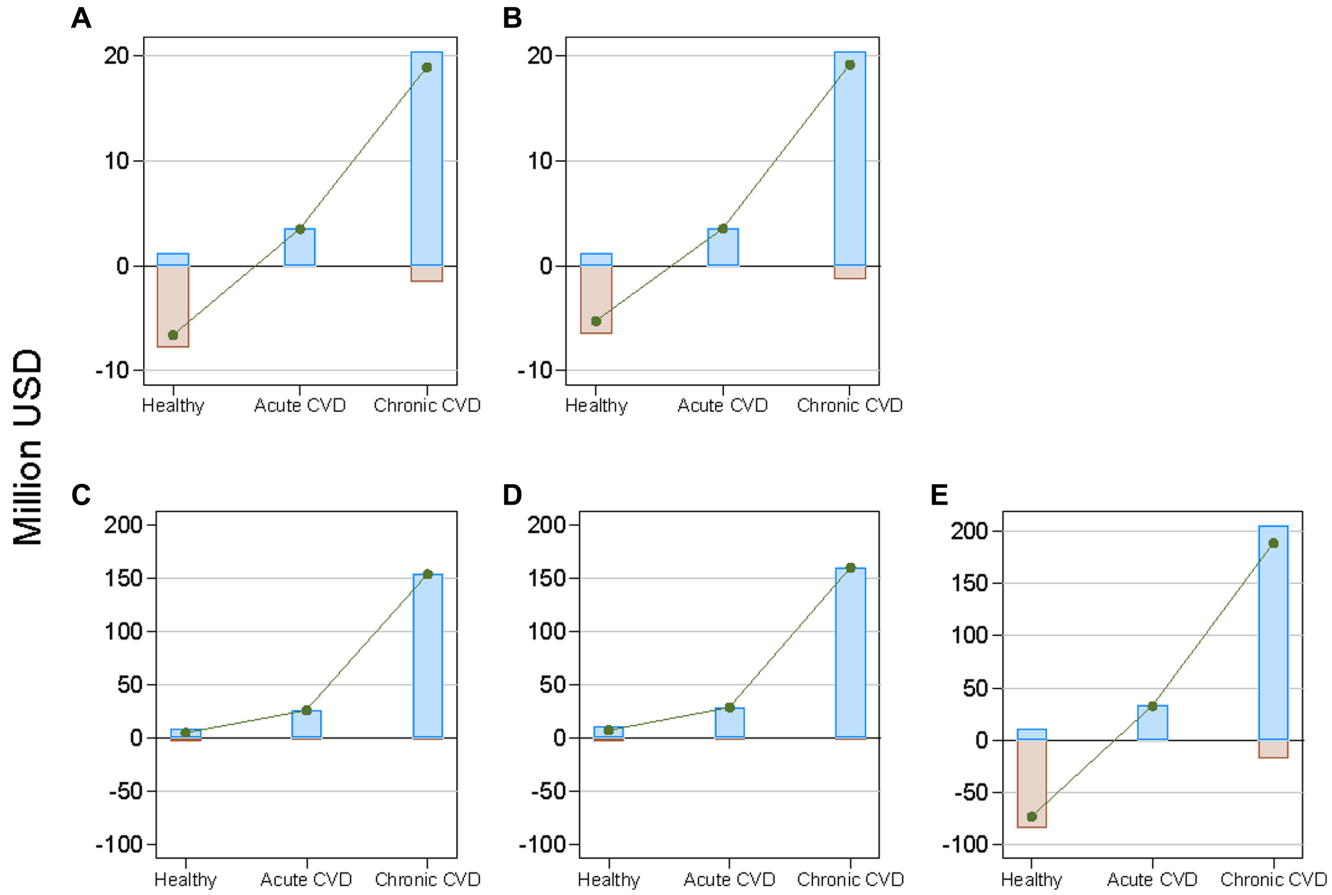
Projected cumulative costs, benefits, and net benefits from reducing mean dietary salt intake between 2019 and 2029, by health state and policy, in a closed cohort of the total population of all ages in 2019. Brown bars indicate costs; blue bars indicate benefits; and green lines indicate net benefits. CVD denotes cardiovascular disease. **(A)**: national media campaign; **(B)**: front-of-pack traffic light labeling; **(C)**: voluntary reformulation; **(D)**: mandatory reformulation with the best-case policy cost; **(E)**: mandatory reformulation with the worst-case policy cost.

By health state, chronic CVD accounted for approximately 80% of the benefits achieved by each policy (Figure 3). The costs were substantially greater than the benefits in the healthy state for all policies, except voluntary reformulation. Consequently, the net benefit was largest for chronic CVD and smallest for being healthy for all policies.

### Sensitivity analyses

**Figure 4:**
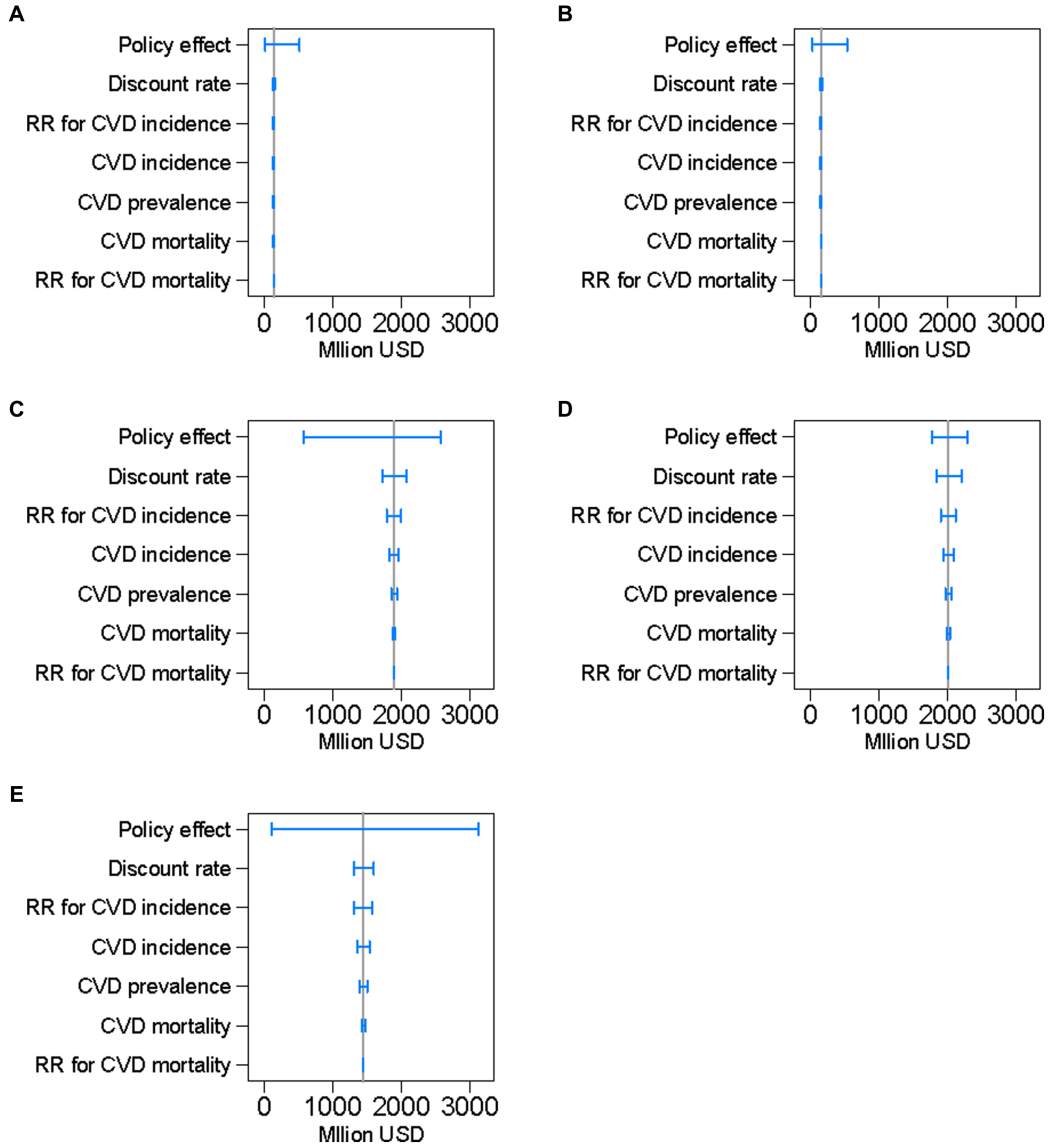
Results of the one-way sensitivity analyses of key input parameters on projected cumulative net benefits of preventing cardiovascular diseases, by policy, between 2019 and 2029 in a closed cohort of the population of all ages in 2019. The blue range plots with spikes indicate uncertainty ranges and the gray vertical lines indicate expected values. CVD denotes cardiovascular disease and RR denotes relative risk. **(A)**: national media campaign; **(B)**: front-of-pack traffic light labeling; **(C)**: voluntary reformulation; **(D)**: mandatory reformulation with the best-case policy cost; **(E)**: mandatory reformulation with the worst-case policy cost.

Figure 4 illustrates the results of the one-way sensitivity analyses. The modeled uncertainty in cumulative net benefits over 10 years was largest for the effects of policies on salt reduction, followed by the discount rate, and the relative risk for CVD incidence.

## Discussion

Our simulation results showed that all the salt reduction policies bring positive net benefits accumulated over 10 years. The majority of the net benefits were attributable to reduced outpatient medical expenses for chronic CVD. The net benefit was far greater for voluntary or mandatory reformulation of processed foods by food manufacturers than for media campaigns and labeling of packaged food. The net benefit of mandatory reformulation was estimated to substantially vary by the policy cost and surpass that of voluntary reformulation when there was no policy cost incurred to the industry. This finding partly supports previous studies showing that mandatory salt reduction policies might be more cost-effective or cost-saving than voluntary strategies (20, 51). Limitations of voluntary strategies have been shown in the United Kingdom (UK), where decreases in mean dietary salt intake slowed after implementation of the Public Health Responsibility Deal based on a voluntary agreement between the government and the food industry (52, 53).

The results of our simulation model based on the hypothetical scenarios do not suggest any decisive direction regarding the effects of salt reduction policies on the reduction of CVD-related healthcare costs in Japan. The significance of our study lies in demonstrating the specific steps for conducting simulation analyses by fitting Japanese data to predict future effects of nutrition policies on the reduction of healthcare expenditures.

In Western countries, including the UK, a major source of salt intake is processed foods such as bread, whereas in Japan and some other Asian countries, more than half of the salt intake comes from discretionary salt intake, or salt added during cooking and at the dining table (54). However, a previous study showed that younger generations in Japan consume a higher proportion of salt from processed foods and eating out than other age groups (55). In future simulation research in Japan, it would therefore be important to include different strategies such as salt substitutes examined in China (12), while continuing to analyze policies for lowering the sodium levels in processed foods as well. In March 2022, the Japan Ministry of Health, Labour, and Welfare has established the “Healthy and Sustainable Food Environment Strategy Initiative” and began collaborating with academia and industry to address nutrition and environmental issues, including excessive salt intake (56). The initiative aims to support food manufacturers and distributors in the development of products and merchandise displays to enable consumers to effortlessly reduce their salt intake. In this social context, it is urgent to establish health economic evaluation methods for nutrition policies, including salt reduction policies in Japan.

Globally, the World Health Organization (WHO) recommended that all member states reduce population salt intake by 30% between 2013 and 2025 (57), but the progress toward achieving this target has been slow despite efforts in an increasing number of countries (58). In 2021, the WHO established global benchmarks for sodium levels in foods across 18 food categories to facilitate reformulation of food products (59). Recently, the WHO published a report using Sodium Country Score Cards, which monitors each country’s progress in making national commitments and taking a multifaceted approach to implementing policies to reduce sodium intake (7). The report listed the following “WHO Best Buys” for preventing non-communicable diseases through reducing sodium intake as voluntary or mandatory measures: a mass media campaign to reduce sodium intake; public food procurement and service policies limiting salt or sodium-rich food; reformulation targets or maximum limits for sodium in food; and front-of-pack nutrition labeling that includes sodium. The scores ranged from 1 (the lowest) for a national policy commitment to reduce sodium intake to 4 (the highest) for multiple mandatory measures adopted for sodium reduction, and implementation of all related WHO Best Buys for tackling non-communicable diseases. The UK was given Score 3 with mandatory measures, specifically public food procurement and service policies. Japan was also given Score 3 with mandatory measures such as labeling of sodium chloride equivalent on all prepackaged food as well as public food procurement and service policies (60). The present study will be useful when Japan prioritizes the WHO Best Buys.

In future research, it is necessary to develop a model that is unique to salt reduction policies in Japan, using the model created in this analysis as a reference. To support this effort, the construction of a database on the effects and costs of programs for salt reduction is an urgent task. We were forced to use data from England for this simulation because virtually no data were available on the costs of Japan’s salt reduction policies. As a rare case, the budgets of a prefectural movement for salt reduction conducted from 2009 to 2018 were reported (e.g., approximately 6.2 million yen in 2016 and 6.3 million yen in 2017), but no further details on costs were disclosed (61).

Our study has limitations to note. First, our model is based on scenarios and data on salt reduction policies in England. Regarding the front-of-pack nutrition labelling, we examined only the traffic-light system and did not consider other effective schemes such as nutrient scores and warnings (62). Second, our model simulated total population and did not consider differences by sex and age in the levels of salt intake, epidemiological indicators of CVD, and medical expenses. Third, for simplicity, our model was based on the overall effect of salt reduction on CVD that included the intermediary effect of blood pressure. As the relationship between salt intake and blood pressure is well established (63), future models should explicitly incorporate blood pressure. Fourth, to simplify the model, we also assumed that the full effect of dietary salt reduction on CVD and stroke was achieved immediately without any phase-in period. It should be considered in future research that the effects on CVD outcomes may take longer to be observed. Fifth, the cost for voluntary reformulation includes only the monitoring cost, which might not necessarily be the case with Japan not having launched any policies for reducing salt content in processed foods. Voluntary approaches might not have the same costs as those for reformulation within natural product cycles. Finally, we defined the benefits as the reduced medical expenses for CVD, but did not consider disabilities and quality of life among survivors and potential increases in medical expenses for non-CVD diseases throughout the life extended by prevention of CVD.

In conclusion, as part of efforts to predict the future savings of social security costs related to nutrition policies in Japan, we created a simulation model of hypothetical scenarios involving the implementation of salt reduction policies used in England with published data on policy costs and effects. Japanese data on cardiovascular epidemiology and healthcare spending were partially incorporated to perform the simulation analyses. Thus, we demonstrated a reference model for conducting a simulation analysis by applying Japanese data. In future studies, it is necessary to refine the model for Japan while establishing a database network on the costs and effects of domestic salt reduction policies.

## Data availability statement

The original contributions presented in the study are included in the article/Supplementary material, further inquiries can be directed to the corresponding author.

## Author contributions

NI and NN contributed to conception and design of the study and organized the database. NI, HY, and NN contributed to the acquisition and interpretation of data. NI, JH, HK, and NN performed the simulation analysis. NI wrote the first draft of the manuscript. All authors contributed to manuscript revision, read, and approved the submitted version.
