# Supplementary material for: Economic effects of dietary salt reduction policies for cardiovascular disease prevention in Japan: a simulation study of hypothetical scenarios
Source: Front Nutr. 2023 Nov 9;10:1227303. doi: 10.3389/fnut.2023.1227303 (PMC10665469; doi:10.3389/fnut.2023.1227303)
Supplement: Supplementary file 1 [file Data_Sheet_1.pdf]

Supplementary Table 1. Distribution of the cohort members among health states at the start of the simulation

	Health state	Proportion
P_1	Healthy	$1 – P_2 – P_3$
P_2	Acute CVD	CVD incidence rate / 100,000
P_3	Chronic CVD	CVD prevalence rate / 100,000
P_4	Dead	0

CVD, cardiovascular disease.

Supplementary Table 2. Initial values of state transition probabilities

	Start	End	Formula
P_{11}	Healthy	Healthy	$1 – P_{12} – P_{14}$
P_{12}	Healthy	Acute CVD	$(P_2 – P_3 * P_{32}) / P_1$
P_{14}	Healthy	Dead	(Total deaths / Total population – CVD mortality rate / 100,000) / P_1
P_{23}	Acute CVD	Chronic CVD	1 – CVD mortality rate / CVD incidence rate
P_{24}	Acute CVD	Dead	CVD mortality rate / CVD incidence rate
P_{32}	Chronic CVD	Acute CVD	0.04
P_{33}	Chronic CVD	Chronic CVD	$1 – P_{32} – P_{34}$
P_{34}	Chronic CVD	Dead	0.02

CVD, cardiovascular disease.

Supplementary Table 3. Mean salt intake (g/day) and cumulative risk reductions in cardiovascular (CVD) incidence and mortality in each year

	2019	2020	2021	2022	2023	2024	2025	2026	2027	2028
National media campaign and front-of-pack traffic light labelling										
Mean salt intake	10.080	10.059	10.039	10.019	9.998	9.978	9.958	9.938	9.918	9.898
CVD incidence	0.992	0.985	0.978	0.970	0.963	0.956	0.949	0.942	0.935	0.928
CVD mortality	0.965	0.932	0.899	0.868	0.838	0.809	0.782	0.755	0.729	0.704
Voluntary reformulation										
Mean salt intake	9.937	9.777	9.619	9.464	9.312	9.162	9.014	8.869	8.726	8.585
CVD incidence	0.940	0.884	0.832	0.785	0.740	0.699	0.661	0.625	0.592	0.562
CVD mortality	0.722	0.525	0.384	0.282	0.209	0.155	0.116	0.087	0.066	0.050
Mandatory reformulation										
Mean salt intake	9.877	9.659	9.446	9.238	9.034	8.834	8.639	8.449	8.262	8.080
CVD incidence	0.916	0.844	0.777	0.717	0.663	0.614	0.570	0.530	0.493	0.460
CVD mortality	0.620	0.451	0.331	0.244	0.181	0.135	0.101	0.076	0.058	0.044

Supplementary Table 4. Calculation of costs of salt reduction policies used in the analysis

Policy	Costs for 10 years, GBP*			Annual total costs, USD†
	Policy cost	Monitoring cost	Total	
National media campaign	50,039,670	0	50,039,670	12,774,263
Front-of-pack traffic light labelling	20,000,000	21,461,538	41,461,538	10,584,414
Voluntary reformulation	0	21,461,538	21,461,538	5,478,760
Mandatory reformulation with the best-case policy cost	0	21,461,538	21,461,538	5,478,760
Mandatory reformulation with the worst-case policy cost	500,000,000	21,461,538	521,461,538	133,120,121

* The costs for 10 years were obtained from Collins, et al (2014)[1]. The policy cost for mandatory reformulation with the worst-case policy cost was estimated as the product of 25,000 GBP per processed food product for salt reduction and the target of 20,000 product lines. The policy cost for front-of-pack traffic light labelling was estimated as the product of the average cost of labelling (1,000 GBP per stock control unit) and 20,000 product lines needed to change labels to a signaling system.

† The total costs in GBP were doubled, because the total population of Japan was approximately twice that of England and Wales. The doubled costs were further divided by 10 to obtain annual costs. The annual costs in GBP were then converted to USD according to the annual average exchange rate in 2019 (0.783 GBP per USD).[2]

References

1. Collins M, Mason H, O'Flaherty M, Guzman-Castillo M, Critchley J, Capewell S. An economic evaluation of salt reduction policies to reduce coronary heart disease in England: a policy modeling study. Value Health. 2014;17(5):517-24.
2. International Monetary Fund. IMF data access to macroeconomic & financial data [February 4, 2022]. Available from: https://data.imf.org/?sk=388dfa60-1d26-4ade-b505-a05a558d9a42.
